# Functional characterization of AVPR2 mutants found in Turkish patients with nephrogenic diabetes insipidus

**DOI:** 10.1530/EC-17-0236

**Published:** 2017-11-08

**Authors:** Beril Erdem, Angela Schulz, Emel Saglar, Ferhat Deniz, Torsten Schöneberg, Hatice Mergen

**Affiliations:** 1 Department of BiologyFaculty of Science, Hacettepe University, Ankara, Turkey; 2 Rudolf Schönheimer Institute of BiochemistryFaculty of Medicine, Leipzig University, Leipzig, Germany; 3 Department of EndocrinologySBÜ Sultan Abdülhamid Han Teaching Hospital, Istanbul, Turkey

**Keywords:** AVPR2, diabetes insipidus, GPCR, functional analysis

## Abstract

Diabetes insipidus is a rare disorder characterized by an impairment in water balance because of the inability to concentrate urine. While central diabetes insipidus is caused by mutations in the *AVP*, the reason for genetically determined nephrogenic diabetes insipidus can be mutations in *AQP2* or *AVPR2*. After release of AVP from posterior pituitary into blood stream, it binds to AVPR2, which is one of the receptors for AVP and is mainly expressed in principal cells of collecting ducts of kidney. Receptor activation increases cAMP levels in principal cells, resulting in the incorporation of AQP2 into the membrane, finally increasing water reabsorption. This pathway can be altered by mutations in *AVPR2* causing nephrogenic diabetes insipidus. In this study, we functionally characterize four mutations (R68W, ΔR67-G69/G107W, V162A and T273M) in AVPR2, which were found in Turkish patients. Upon AVP stimulation, R68W, ΔR67-G69/G107W and T273M showed a significantly reduced maximum in cAMP response compared to wild-type receptor. All mutant receptor proteins were expressed at the protein level; however, R68W, ΔR67-G69/G107W and T273M were partially retained in the cellular interior. Immunofluorescence studies showed that these mutant receptors were trapped in ER and Golgi apparatus. The function of V162A was indistinguishable from the indicating other defects causing disease. The results are important for understanding the influence of mutations on receptor function and cellular trafficking. Therefore, characterization of these mutations provides useful information for further studies addressing treatment of intracellularly trapped receptors with cell-permeable antagonists to restore receptor function in patients with nephrogenic diabetes insipidus.

## Introduction

Water homeostasis of the body is rigidly controlled by several mechanisms. Various sensor systems such as endothelial baroreceptors and hypothalamic osmoreceptors, which detect reduced blood volume (hypovolemia) and increased blood electrolyte concentration (hypernatremia), respectively, are capable to sense changes in water balance and serum osmolality. Reduction in blood volume or increase in blood electrolyte concentration induces the release of arginine vasopressin (AVP) from the posterior pituitary gland. AVP is transported to the kidneys via the bloodstream and binds to one of its receptors, the arginine vasopressin type 2 receptor (AVPR2). The hormone–receptor complex initiates the activation of the Gs protein resulting in the activation of the adenylyl cyclase and an increase of the intracellular cyclic AMP (cAMP) concentration. Elevated cAMP levels promote the phosphorylation of aquaporin type 2 water channels (AQP2) by protein kinase A (PKA), which redistributes AQP2 from intracellular vesicles to the apical plasma membrane. Because of this redistribution, water permeability of the cell membrane of principal cells is changed and water reabsorption occurs from the pro-urine into the medullary interstitium following an osmotic gradient (1, 2). Disruption of the renal AVPR2/AQP2 pathway can cause nephrogenic diabetes insipidus (NDI) characterized by the inability to concentrate urine (3, 4). Patients have abnormally diluted and large volumes of urine. Undiagnosed NDI in newborns can also have other early symptoms including vomiting and anorexia, failure to thrive, fever and constipation (5). Furthermore, side effects of some drugs, electrolyte disturbances or urinary tract obstruction can cause an acquired type of NDI (6). However, mutations in *AVPR2* and/or *AQP2* are the main causes of inherited NDI. Because of the mutated gene, NDI can be X-linked (*AVPR2*) or autosomal recessive or dominant (*AQP2*). The vast majority of genetic causes of NDI is based on mutations in the *AVPR2*, which is located on Xq28 (3). The coding sequence of *AVPR2* is distributed on three exons encoding a protein of 371 amino acids. As a member of the superfamily of G protein-coupled receptors, the protein has seven transmembrane helices, three extracellular and three cytoplasmic loops (7). The properties of folding and trafficking of the receptor to the cell membrane may be affected according to the site and/or influence of the mutation on receptor function. Therefore, it is important to analyze the impact of a given mutation on receptor function. In this study, we characterized the functional properties of mutations in the *AVPR2* (R68W, ΔR67-G69/G107W, V162A and T273M), which were previously described in one of our studies (8).

## Materials and methods

### Case history

In our previous study, we presented mutations in the *AVPR2*, *AQP2* and *AVP* genes. Some of these mutations were not described before (8). For the *AVPR2* gene, we identified R68W, ΔR67-G69/G107W, V162A and T273M mutations in four male patients from different, unrelated families. They were diagnosed with NDI after they had referred to Gulhane Military Medical Academy, Department of Endocrinology and Metabolism. In Turkey, almost all young males who show symptoms of some disorders refer to this hospital for evaluation of their eligibility for military service. That is why they were diagnosed with NDI when they referred to the hospital instead of when they were infant. Detailed clinical data of the patients were mentioned in our previous study (8). In the family of the patient harboring the ΔR67-G69/G107W mutation, the uncle and aunt have only the deletion mutation ΔR67-G69. The missense mutation G107W was seen in the patient for the first time. R68W and V162A mutations were just seen in the patients, not in their family members. For the patient who harbors T273M mutation, we could not reach family members to screen for a familial origin.

### Construction of mutant AVPR2 plasmids

All mutants were generated with a PCR-based site-directed mutagenesis and restriction fragment replacement strategy by using a human *AVPR2*, in the mammalian expression vector pL as template. The pL vector is a modified version of the former described pcD-ps vector with a removed poly-A fragment 3′ of the MSC-cloning region (9). The correctness of the mutant V2 receptor plasmids was verified by sequencing. For receptor detection by ELISA, wild-type (WT) and all mutant AVPR2-coding sequences were N- and C-terminal epitope-tagged (N-terminal: hemagglutinin (HA)-tag; C-terminal: FLAG-tag) (10, 11). For immunofluorescence studies, all mutants and the WT receptor were fused to the EGFP sequence right after the AVPR2 coding sequence and before the stop codon. After restriction digest of the plasmids, Gibson assembly method was used to generate constructs with EGFP.

### Cell culture and transfection studies

For cell culture studies, COS-7 cells were grown in DMEM contains 10% FBS, 100 U/mL penicillin and 100 μg/mL streptomycin at 37°C in a humidified 5% CO_2_ incubator. Lipofectamine (Invitrogen) and MACSfectin Reagent (Miltenyi Biotec, Bergisch Gladbach, Germany) were used as agents for transiently transfection of COS-7 cells. For the functional assays, 96-well plates (15 × 10^3^ cells/well), 48-well plates (4 × 10^4^ cells/well), 12-well plates (20 × 10^4^ cells/well) and 6-cm cell culture dishes (70 × 10^4^ cells/well) (cAMP assay, cell surface ELISA, immunoblotting, sandwich ELISA and immunofluorescence studies, respectively) were used. For transfection of 96-well plates, 48-well plates, 12-well plates and 6-cm cell culture dishes, 150 ng DNA to 2 μg DNA were used. The efficiency of transient transfection was checked by EGFP-plasmid transfected cells as transfection control.

### ELISA studies

The cell surface expression of N-terminal HA-tagged mutants was estimated by direct cellular ELISA. To measure the total expression of full-length double-tagged AVPR2s (N-terminal HA-tag, C-terminal FLAG-tag), a sandwich ELISA was used. Both methods of ELISA were performed as described in earlier studies (10, 12, 13).

### cAMP assay

A non-radioactive cAMP accumulation assay based on the ALPHAScreen technology according to the manufacturer’s protocol (Perkin Elmer LAS) was used to determine the cAMP content of the cell extracts. The protocol was performed as it was described before (10, 11). cAMP accumulation data were analyzed using GraphPad Prism, version 5.01 for Windows (GraphPad Software).

### Immunofluorescence experiments

For imaging mutant and WT AVPR2s, COS-7 cells were transfected with the EGFP-tagged constructs. By this way, expressed AVPR2s were detected as green-fluorescent molecules. Labeling of the endoplasmic reticulum (ER) and Golgi apparatus was performed separately by co-transfection with pDsRed2-ER and pDsRed-Monomer-Golgi (Clontech), respectively. 72 h after transfection, COS-7 cells previously seeded on cover slips, were fixed and mounted on glass slides. Images were taken with a confocal laser-scanning microscope (LSM 510; Carl Zeiss Jena Gmbh).

## Results

**Figure 1:**
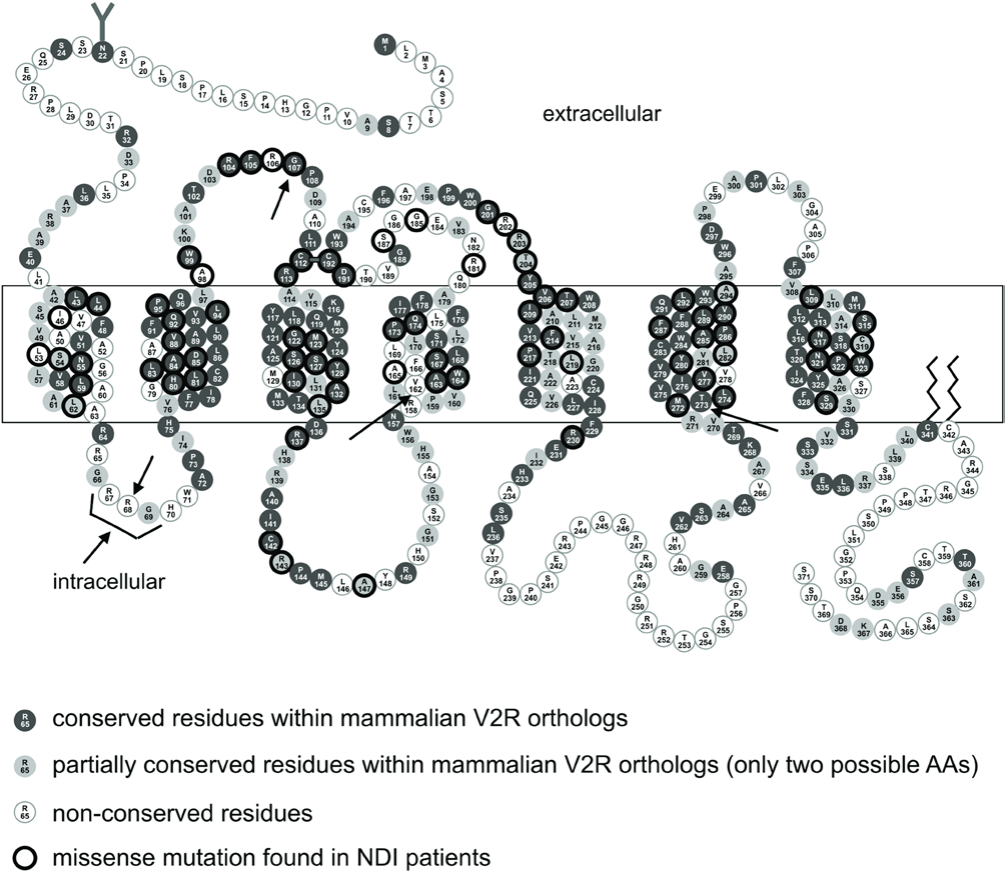
Amino acid sequence and topology of the human AVPR2 (The figure is an excerpt from Böselt *et al*., 2009). Mutations, analyzed in this study are shown with arrows (ΔR67-G69, R68W, G107W, V162A and T273M).

The positions of the mutated amino acids within the receptor are depicted in Fig. 1. Four different mutations were introduced into pLV2R with a PCR-based site-directed mutagenesis and restriction fragment replacement method (R68W, ΔR67-G69/G107W, V162A and T273M). For the ΔR67-G69/G107W mutation, we also generated two constructs (ΔR67-G69 and G107W) to analyze both mutations separately.

**Figure 2:**
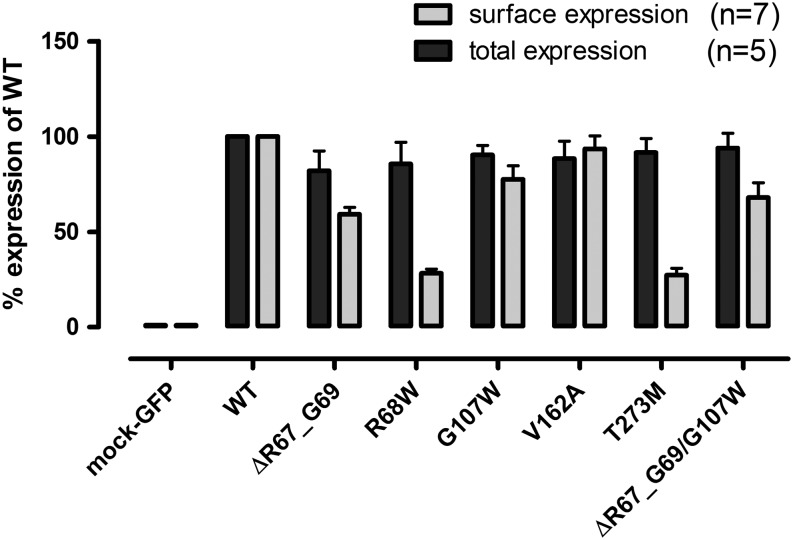
ELISA results of the mutant receptors. COS-7 cells were transfected with WT and mutant AVPR2 plasmids. For the surface expression of WT and mutant AVPR2s, cells were not permeabilized and HA-tagged AVPR2s were detected with peroxidase-labeled monoclonal anti-HA antibody. For detection of total expression of WT and mutant receptors transfected cells were permeabilized. Double-tagged WT and mutant receptors in the cell lysates were detected in a sandwich ELISA using a polyclonal anti-FLAG antibody and peroxidase-labeled monoclonal anti-HA antibody. ‘n’ defines counts of independent experiments.

The functional characterization of the mutant receptors by total and surface ELISA revealed that all mutant receptors were properly synthesized within the cells (Fig. 2; Table 1). However, except of V162A, all mutant receptors were not properly delivered to the cell surface (Table 1). For R68W and T273M, we found only ~28% cell surface expression compared to the WT AVPR2. The double mutation ΔR67-G69/G107W showed 68% of the WT AVPR2 cell surface expression most probably because ΔR67-G69 mainly contribute to the retention (59.3%), whereas G107W was only slightly retained (77.6%) (Fig. 2).

**Table 1:** ELISA and cAMP accumulation assay results of the mutant receptors^a^ .

	**E_max_**(% wt)			**EC_50_**(nM)			**Surface expression** (% wt)			**Total expression** (%wt)		
	Mean	**s.d.**	**s.e.m.**	Mean	**s.d.**	**s.e.m.**	Mean	**s.d.**	**s.e.m.**	Mean	**s.d.**	**s.e.m.**
WT	100.0 (9)			0.4 (9)	0.29	0.01	100.0 (7)			100.0 (5)		
ΔR67-G69	81.7 (9)	32.4	10.8	0.39 (8)	0.23	0.08	59.3 (7)	9.4	3.6	82.0 (5)	23.2	10.4
R68W	47.8 (9)	23.8	7.9	0.28 (9)	0.15	0.05	28.3 (7)	6.0	2.3	85.6 (5)	25.4	11.4
G107W	70.2 (9)	41.7	14.7	43.0 (8)	30.0	10.0	77.6 (7)	18.6	7.0	90.4 (5)	11.0	4.9
V162A	94.6 (9)	29.7	17.1	0.31 (7)	0.32	0.12	93.6 (7)	17.9	6.8	88.4 (5)	20.5	9.2
T273M	44.9 (9)	14.1	8.1	31.0 (3)	30.0	18.0	27.3 (7)	9.5	3.6	91.6 (5)	16.3	7.3
ΔR67-G69/G107W	Incalculable			Incalculable			68.0 (7)	20.5	7.8	94.0 (5)	15.4	7.7

^a^Cell surface ELISA, sandwich ELISA and cAMP accumulation assay were performed as described in the *Materials and methods* section. For all experiments, COS-7 cells were transiently transfected with WT and mutant AVPR2s. The numbers in the parentheses indicate independent experiments the data are given as mean ± s.e.m. of independent experiments. For all experiments, except the EC_50_ values the WT was set to 100% as a reference.

**Figure 3:**
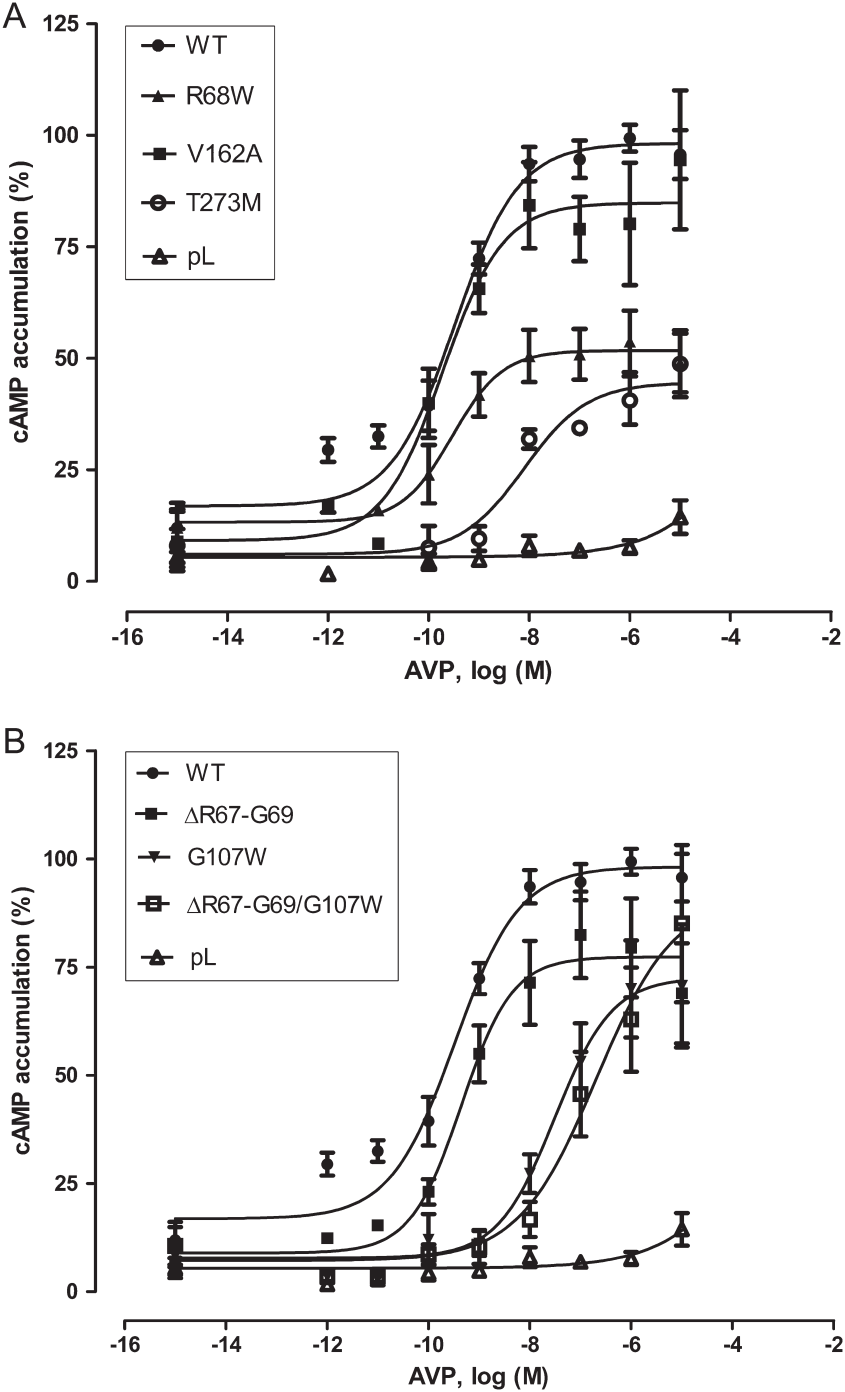
Concentration–response curves of WT and mutant receptors. COS-7 cells were transiently transfected and stimulated with various AVP concentrations as 10-fold dilution series (100 pM–10 μM). AlphaScreen cAMP accumulation assays were done to determine intracellularly cAMP levels in cell extracts. The results were graphed separately. (A) Shows WT, R68W, V162A, T273M and pL. (B) Shows WT, ΔR67-G69, G107W, ΔR67-G69/G107W and pL. GraphPad Prism was used for graphical design of AVP concentration-response curves. All independent experiments were performed in duplicate.

After stimulation of transfected COS-7 cells with increasing concentrations of AVP, mutant receptors showed reduced E_max_ values according to the WT receptor (100%) (Fig. 3A and B and Table 1). EC_50_ values of some mutant receptors were not different from the WT receptor (R68W, ΔR67-G69 and V162A). Mutation of G107W and T273M resulted in a 100-fold higher EC_50_ value compared to WT receptor. For the double mutant ΔR67-G69/G107W, an EC_50_-value and E_max_-value could not be calculated because of an unsaturated curve up to 10 μM. E_max_ values were also reduced for G107W (70.2%) and T273M (44.9%). For R68W mutation, which displayed an unchanged EC_50_ value, E_max_ was reduced to 47.8% of WT receptor. Only the deletion mutation alone (ΔR67-G69; 81.7%) and V162A (94.6%) showed almost WT E_max_ values (Table 1).

**Figure 4:**
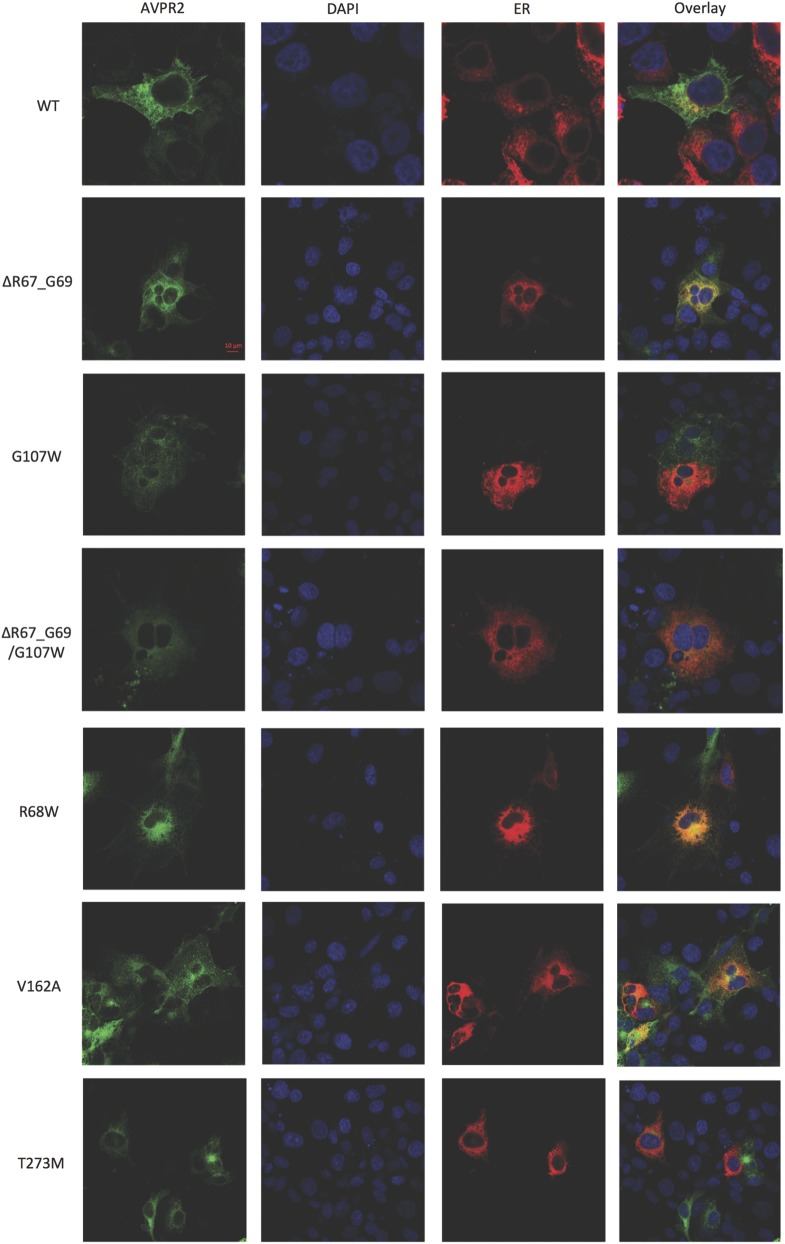
Immunofluorescence studies of AVPR2 mutants and the ER. For visualizing of the sub-cellular localization of WT and mutant AVPR2s, COS-7 cells were grown on glass cover slips and transfected with EGFP-tagged AVPR2 constructs (WT and mutants, green). Nuclei were stained with DAPI (blue). Staining of the ER was performed by co-transfection with pDsRed2-ER (red). Yellow-orange color shows overlay of ER and receptor expression, indicating co-localization of expressed AVPR2 constructs with ER.

Using epitope-tagged WT and mutant AVPR2s to visualize where the intracellularly retained receptors accumulate, immunofluorescence studies were performed (Figs 4 (ER) and 5 (Golgi apparatus)). Comparing WT receptor expression with mutant receptors, most mutants showed reduced surface expression and retention in the ER. The V162A mutant receptor was not found different from WT receptor. Some retention at the Golgi apparatus was seen in all mutants and also in the WT receptor. This could be due to the used ‘overexpression system’ of COS-7 cells.

**Figure 5:**
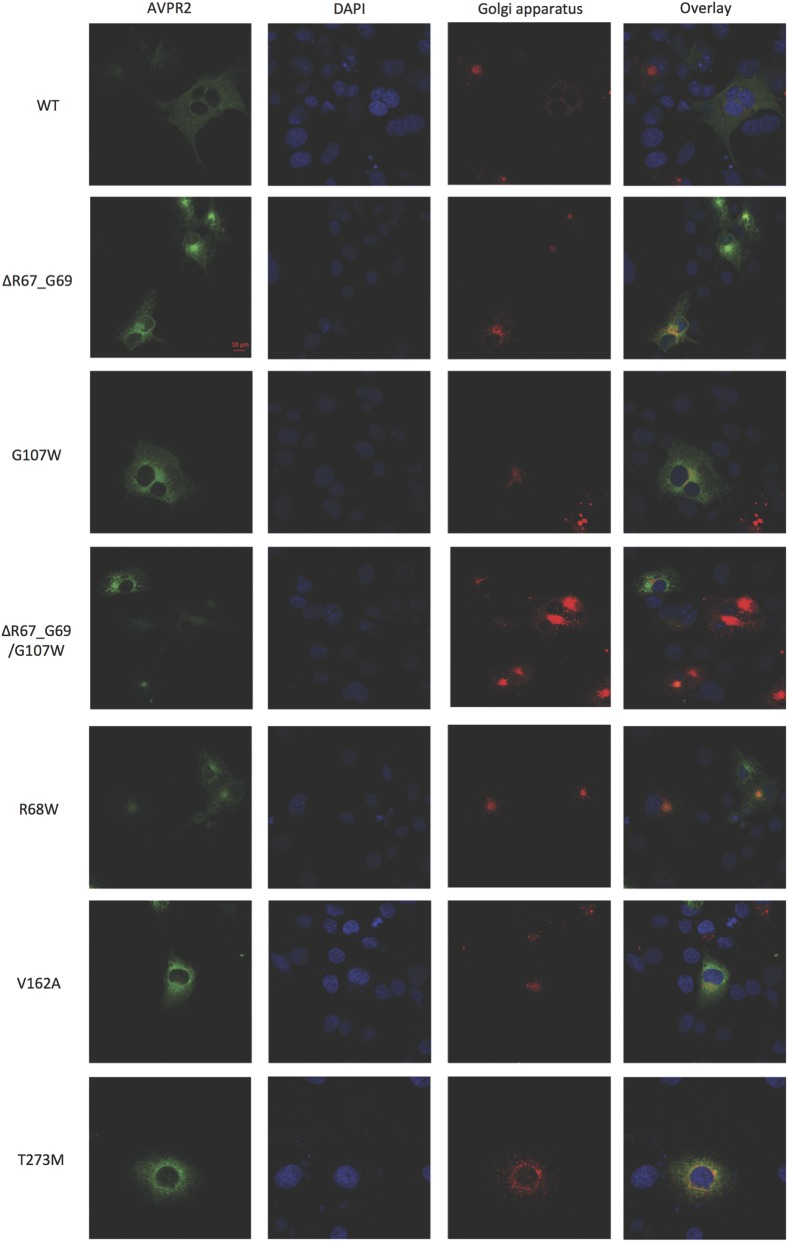
Immunofluorescence studies of AVPR2 mutants and Golgi apparatus. Procedure was the same as described in Fig. 4 for staining of the Golgi apparatus and AVPR2s. COS-7 cells were grown on glass cover slips were transfected with EGFP-tagged AVPR2 constructs (WT and mutants, green). Nuclei were stained with DAPI (blue). Golgi apparatus was co-transfected with pDsRed-Monomer-Golgi (red). Yellow-orange color shows overlay of Golgi apparatus and receptor expression, indicating co-localization of expressed AVPR2 constructs with Golgi apparatus.

## Discussion

Exclusive molecular genetics analysis of the patients’ *AVPR2* gene is not sufficient to support the diagnosis of NDI. The detection of a missense mutation gives a hint for a possible cause of the disease, but functional analysis of the mutated receptor is necessary to verify the impact of the mutation on receptor function. Depending on the functional outcome, there exist some possible therapeutic strategies for specific functional defects. For instance, with chaperone-like antagonists in hand, intracellularly trapped receptors could be rescued to the plasma membrane (14, 15, 16). Another therapeutic approach is usable for NDI patients with up to 10-fold right-shifted concentration–response curves. Here, treatment with higher doses of desmopressin can rescue water reabsorption in the patient’s kidney (3, 12, 17, 18, 19). Therefore, functional characterization studies of *AVPR2* mutations (and also *AQP2* and *AVP*) are important for the treatment strategy of DI patients.

According to the Human Gene Mutation Database Professional 2016.3, the number of mutations found for the *AVPR2* gene is 277. Most of these mutations are missense mutations. In our study, R68W, ∆R67-G69/G107W, V162A and T273M were functionally characterized. Since a mutation may have variable effects on folding and/or trafficking or hormone binding or activation of the V2 receptor, functional characterization studies provides detailed and valuable information.

The missense mutation R68W and the 9 bp deletion (ΔR67-G69) are located in the first intracellular loop (ICL1) of the receptor (Fig. 1). The 9-bp deletion belongs to a repeat sequence resulting in the amino acids RRGRRG in the WT receptor. Deletion of 9 nucleotides from the second RRG-sequence leaves a cytosine from the arginine 67 codon, which is fused to the second and third nucleotide of the histidine 70 codon, resulting again in a codon for histidine (CAC). The two arginine residues (amino acids 67, 68) are not conserved residues in mammalian orthologs (Fig. 1). Codon 69 is a partially conserved residue in mammalian AVPR2 sequences where only 2 different amino acids were found in mammalian othologs. The deletion reduces the length of the short first intracellular loop further. Both can contribute to an improper folding of the receptor reducing the surface expression of this mutant (Fig. 2; Table 1). Our data are in congruence with previous mutagenesis study highlighting the ICL1 as important for cellular trafficking (20). Nevertheless, the functional properties (E_max_ and EC_50_) of the fraction of this mutant receptor, which makes it to the cell surface were comparable to the WT receptor (Table 1).

For R68W, the substitution of arginine at position 68 with tryptophan causes a change from a basic residue to a hydrophobic, bulky residue in a cytosolic environment. Therefore, a change of a residue in terms of hydrophilic and hydrophobic properties can affect receptor folding and function, which can be seen in the markedly reduced surface expression and the E_max_ values of this mutant (Table 1). The reduced functional properties of this mutant could explain why the patient suffers from NDI.

In the receptor protein, amino acid position 107 is located in the second extracellular loop. This position is highly conserved in mammalian AVPR2 sequences, implicating that any change at this codon could affect receptor function. For example, a similar study showed that mutation G107R affected cell surface expression (52.3% ± 6.4 compared to WT receptor) of the protein as well as its E_max_ (67.7% ± 5.5) and EC_50_ (156.2 nM ± 57.0) values (10). It was shown that G107R mutant receptor was synthesized in the cell but could not reach cell surface. For our mutation, G107W, the substitution from glycine, the smallest amino acid to tryptophan, an amino acid with a large hydrophobic side chain is a dramatic change in size and properties. The outcome on receptor function is similar to the G107R mutation regarding surface expression and receptor activation. In our patient, G107W mutation was combined with the 9-bp deletion, resulting in a double mutated receptor protein. Combination of both mutations resulted in a reduced cell surface expression probably due to ER retention of the misfolded protein (Table 1). Additionally, E_max_ and EC_50_ values could not be estimated because of the more than 100-fold right-shifted concentration–response curve (Table 1).

The mutation of valine 162 to alanine had no measurable effect on receptor expression and function (Table 1). Both amino acids are nonpolar and the codon 162 is not conserved among the mammalian orthologs (Fig. 1). In functional studies, V162A mutant showed no significant difference from the WT receptor (Table 1). However, the patient harboring the V162A mutation produces approximately 12 L urine daily, which is a typical symptom of NDI, and it was also reported, that the symptoms have been seen since his childhood. We sequenced all exons and intron–exon boundaries of *AVPR2*, *AQP2* and *AVP* genes but we did not find any mutation at these sites. For this patient, we could not find a causal correlation between mutation and symptoms.

In contrast to V162A, the mutation T273M affects a highly conserved residue in the transmembrane helix 6 (TMD6). This implicates, that this position, close to the cytoplasm, is likely to be important for proper receptor function. Numerous studies have shown, that during receptor activation position of TMD6 is moving toward the cytoplasm and residues were exposed to the cytosol (21, 22, 23). The substitution of threonine 273 to methionine changes a polar to a nonpolar amino acid side chain. Even though the T273M mutant protein was well expressed in the cell (91.6%), surface expression was severely reduced (27.3%) compared to the WT receptor (Table 1). cAMP accumulation assays indicated a 77.5-fold increase in the EC_50_ value with a maximal response of 44.9% compared to the WT receptor (Table 1). The impact of these mutations on the receptor function doubles explains the patient’s NDI phenotype.

Most mutations in AVPR2 result in receptor misfolding (21) eventually leading to quality control of these mutant proteins in ER or Golgi apparatus (24). Our immunofluorescence studies and the surface ELISA results support this notion. We found that some of our mutants, especially the mutants which have a severe phenotype (R68W, ΔR67-G69/G107W and T273M), could not properly reach the cell surface because of a retention in the ER. Therefore, rescuing the misfolded proteins from retention mechanisms by using chemical chaperons can have beneficial effects and could help in treating the disease (6, 25).

NDI is a disorder that affects daily life of the patients due to the production of large volumes of urine, even at nights. Therefore, early diagnosis and treatment of the patient are pivotal to make patients’ life better (1). Early diagnosis and the developing of new treatment strategies are strongly associated with functional studies of mutant proteins, as in the present study.

## Declaration of interest

The authors declare that there is no conflict of interest that could be perceived as prejudicing the impartiality of the research reported.

## Funding

This study was supported by The Scientific and Technological Research Council of Turkey (Project number: 112S513) and Beril Erdem was supported by an EMBO Short Term Fellowship (Place of Fellowship: University Leipzig, Leipzig, Germany, ASTF No: 255-2013).
